# Advancing with the 2024 Latin America and the Caribbean Periodontal Consensus

**DOI:** 10.1590/1807-3107bor-2024.vol38.0115

**Published:** 2024-11-22

**Authors:** Claudio Mendes PANNUTI, Cristina Cunha VILLAR

**Affiliations:** (a) Universidade de São Paulo – USP, School of Dentistry, Department of Periodontics, São Paulo, SP, Brazil.

The first Latin America and the Caribbean Periodontal Consensus, held in 2020,^1^ marked a historical milestone in the dentistry of the region. Planned before publication of the 2017 Classification of Periodontal and Peri-Implant Diseases and Conditions,^2^ this consensus brought together experts from across Latin America and the Caribbean to address the unique challenges faced in the region, and its characteristics in managing periodontal diseases. It was a pioneering effort with the aim of creating a common foundation of understanding and practices for a diverse population, reflecting the region’s epidemiological, socioeconomic, and cultural realities.

However, the evolution of scientific knowledge and the release of new international guidelines, such as the 2017 Classification of Periodontal and Peri-Implant Diseases^2^ and the 2020 EFP S3-level Clinical Practice Guidelines,^3^ highlighted the need for a novel consensus. These guidelines introduced significant advances in the understanding and management of periodontal diseases. Consequently, the 2024 Consensus not only updated its recommendations to align with these advances but also adapted these global guidelines to address the specific needs and challenges of our region, ensuring that periodontal practice in Latin America and the Caribbean remains informed about the latest scientific evidence, in addition to tailoring it to the local context.

By addressing the critical aspects of periodontal disease management, the 2024 Consensus covers a wide range of topics, from prevalence, burden, and risk factors through to diagnosis, prevention, and treatment.^4-9^ In a region where the per capita health expenditure is approximately $650, significantly lower than the global average of $1,300, according to the World Health Organization Global Health Expenditure database (2024),^10^ and considering that dental expenditure in low-income countries is 500 times lower than it is in high-income countries,^11^ the consensus underscores the urgent need to optimize both financial and human resources. It is crucial for treatment protocols to be based on the most reliable and current scientific evidence. The consensus advocates for a strategic approach that prioritizes cost-effective, primary care-based interventions, including prevention, early diagnosis, and evidence-based treatments. These strategies have the potential to reduce overall healthcare costs, enhance public health outcomes, and address health inequalities.^12,13^

In this context, the 2024 Consensus highlighted the prevalence of periodontal diseases in Latin America and the Caribbean, as revealed by a comprehensive review of regional studies.^4^ This review, which analyzed data from 35 studies across 12 countries, emphasized the variability in reported prevalence rates, influenced by factors such as age, methodology, and case definitions. Despite the recent increase in studies with representative samples, the lack of methodological consistency prevents a unified conclusion. This underscores the need for multicenter studies with standardized protocols to enable better understanding and addressing of the region’s periodontal disease burden. Consequently, the Consensus recommends collaborative efforts with the aim of enabling countries to conduct studies with reproducible methodologies, thereby ensuring the generation of data that accurately reflects the region’s diverse needs and realities.

The 2024 Consensus also delves into the substantial burden and impact of periodontal diseases on both oral health-related quality of life (OHRQoL) and systemic health conditions in Latin America and the Caribbean.^5^ A comprehensive scoping review of the existing research indicated that periodontal diseases, particularly severe periodontitis, are prevalent in the region and negatively impact OHRQoL, contributing to psychological discomfort, and both physical and social disability. Moreover, the review underscored the strong associations between periodontal diseases and various systemic conditions, including diabetes, cardiovascular diseases, and adverse pregnancy outcomes. These findings emphasize the need for integrated healthcare strategies that address both periodontal health and systemic diseases since they reinforce the critical role of periodontal care in improving overall health outcomes in the region.

This Consensus also explored the risk factors contributing to the development and progression of periodontitis in Latin America and the Caribbean by emphasizing the significant role of smoking and diabetes.^6^ A critical review of regionally representative studies revealed a strong association between smoking and periodontitis, particularly among heavy smokers and highlighted smoking as a major risk factor. Although diabetes was also identified as a potential risk factor, the association with periodontitis was less consistent across studies after adjusting for confounding variables. In alignment with a recent consensus by the European Federation of Periodontology,^14^ these findings underscored the need for targeted public health interventions to address smoking cessation and better management of diabetes as part of comprehensive periodontal care strategies in the region. The Consensus calls for more robust, prospective cohort studies to further elucidate these associations and to inform tailored prevention and treatment approaches that address the unique challenges faced by populations in Latin America and the Caribbean

By recognizing the key role of accurate and comprehensive diagnosis, the 2024 Consensus stresses its importance as the cornerstone for effective treatment and management strategies in Latin America and the Caribbean.^7^ Despite advancements in the classification and understanding of periodontal diseases,^2^ the region continues to face significant challenges in diagnosing periodontal diseases and conditions. The underdiagnosis of periodontal diseases, often due to a historical focus on dental caries and the insufficient implementation of diagnostic protocols, remains a concern. The consensus calls for a more rigorous approach, emphasizes the necessity of performing complete periodontal examination and cautions against reliance on partial examination methods since these lead to the risk of underestimating disease prevalence and severity. By prioritizing thorough diagnosis across all levels of care, the 2024 Consensus seeks to enhance treatment outcomes and address the ongoing burden of periodontal diseases throughout Latin America and the Caribbean.

Prevention is another critical focus of the current Consensus, which advocates for integrated public health strategies that address both oral and systemic health.^8^ By incorporating periodontal prevention into broader health promotion programs and addressing shared risk factors such as smoking and poor diet,^15^ the consensus aims to reduce the high burden of periodontal diseases in the region. It also underscores the importance of community-based initiatives and active involvement of dental professionals in empowering patients and promoting self-care. Furthermore, the consensus recognizes the necessity of developing and implementing tailored preventive strategies that consider the region’s diverse socioeconomic and cultural landscape, as well as the unique challenges posed by different life stages.

In alignment with the European Federation of Periodontology (EFP) S3-level clinical practice guidelines,^3^ the 2024 Consensus further advocates for a patient-centered, multifaceted approach tailored to the unique challenges faced by Latin American and Caribbean populations.^9^ This approach spans a continuum of care, from self-care and non-surgical interventions through to surgical treatments, all based on the latest scientific evidence. The consensus highlights the vital role of supportive periodontal care (SPC) in maintaining long-term oral health, particularly by addressing the factors that contribute to non-adherence, which is a significant issue in this region. Moreover, it acknowledges existing oral health policies in several Latin America and Caribbean countries (LACC), such as Brazil’s Family Health Strategy (FHS) and the Dental Specialties Centers (DSCs), while also emphasizing the persistent challenges, including limited access to primary and specialized care services. To address these obstacles, the 2024 Consensus advocates for the enhancement of existing policies to optimize resource allocation and promote equitable access to high-quality periodontal care across the region.

As we move forward, the 2024 Consensus represents a decisive call to action. It challenges all stakeholders—clinicians, policymakers, educators, and the broader healthcare community—to elevate periodontal care by embracing evidence-based practices tailored to the region’s unique realities. By fostering collaboration, prioritizing prevention, and committing to comprehensive and equitable care, we can mitigate the burden of periodontal diseases and enhance the overall health of populations across Latin America and the Caribbean.
